# Abatement of the ecotoxicological risk of landfill leachate by heterogeneous Fenton-like oxidation

**DOI:** 10.1007/s11356-022-23682-6

**Published:** 2022-10-20

**Authors:** Sajid Hussain, Eleonora Aneggi, Clara Comuzzi, Diego Baderna, Daniele Zuccaccia, Alessandro Trovarelli, Daniele Goi

**Affiliations:** 1 Dipartimento Politecnico Di Ingegneria E Architettura, Università Di Udine, Unità Di Ricerca INSTM, Udine, Italy; 2 Dipartimento Di Scienze Agroalimentari, Ambientali E Animali, Università Di Udine, Via Cotonificio 108, 33100 Udine, Italy; 3 Dipartimento Ambiente E Salute, Istituto Di Ricerche Farmacologiche Mario Negri IRCCS, Milan, Italy

**Keywords:** Fenton-like, Leachate, AOP, AOX, ^1^H NMR

## Abstract

Landfill leachates are highly contaminated liquid waste, and their treatment and detoxification are a challenging task. The current system of ecotoxicological risk assessment is complex and time-consuming. It is of fundamental importance to develop simpler and faster tools for the evaluation of the treated liquid waste and for an easier preliminary screening of the most active catalytic formulation/reaction conditions of the Fenton-like process. Here, several analytical techniques have been used for the assessment of the reduction of toxicity of the landfill leachate after Fenton process over copper-zirconia catalyst (ZrCu). Ultraviolet–visible (UV–vis) spectroscopy and absorbable organic halogens (AOX) analysis have been coupled to achieve further insight into the degradation of contaminants. In addition, for the first time, the qualitative abatement of organic compounds is monitored through proton nuclear magnetic resonance (^1^H NMR) analysis, providing a new method for evaluating the effectiveness of the treatment. Spectroscopic techniques reveal that the Fenton process induces a significant abatement of the aromatic and halogen compounds (51%) in the landfill leachate with a reduction of the toxicity that has been confirmed by ecotoxicological test with algae. These results validate the investigated tool for a simple rapid preliminary evaluation of the detoxification efficacy.

## Introduction

Landfill leachate has become a serious environmental concern because it is a heavily polluted liquid waste and consists of contaminants such as organics, inorganics, nitrogen/ammonia, heavy metals etc., that are not effectively degraded by conventional biological technologies (Wiszniowski et al. 2006). Advanced oxidation processes (AOPs) are among the most effective treatment methods for the abatement of refractory pollutants and thus can be applied for the depollution of leachate (Wang & Xu 2012). Indeed, in a recent study, the available treatment techniques for hazardous aniline‑based organic contaminants have been compared, and AOPs were found to be the most promising treatment methods (Chaturvedi 2022). A heterogeneous Fenton process, generally referred to as Fenton-like process, remains the most viable solution for the treatment of liquid waste (Jain et al. 2018; Molina et al. 2020; Nidheesh 2015; Zhu et al. 2019). Recently, in our laboratories we have developed a series of zirconia supported catalysts that have shown promising performance in the treatment of model aqueous solutions of ibuprofen (Hussain et al. 2020, 2021) and in the treatment of landfill leachate (Hussain et al. 2022). Here, on the basis of previous studies, we have selected a copper supported on zirconia catalyst for leachate treatment with the aim of investigating a simple approach and methodology to monitor the ecotoxicological risk reduction in landfill leachate after treatment with heterogeneous Fenton-like oxidation.

The study is mainly focused on the qualitative assessment of the abatement of the organics by means of several techniques, such as UV–vis spectra, absorbable organic halogens (AOX) content and toxicity on unicellular green algae. In addition, to the best of our knowledge, this is the first time that ^1^H NMR spectroscopic analysis has been applied to monitor the qualitative abatement of contaminants from landfill leachate, providing useful information on the nature of organics, with particular attention to the degradation of aromatic compounds and on the effectiveness of the process.

The resonant frequencies of a NMR spectrum of a sample containing active nuclei are dependent, not only on the external magnetic field at the nucleus, but also on the local magnetic field that depends on molecular environment (the nature of the surrounding electrons and atoms). As a result, information about the nucleus and chemical environment can be derived from its resonant frequency, and this is related to the nature of the local electron of the nucleus and of the neighboring atoms. In other words, ^1^H NMR spectroscopy can be efficiently used to characterize functional groups present in an organic molecule and permit to determine the structure of a target substances. ^1^H NMR is a valuable analytical tool for the characterization of chemical compounds and has been widely used for the characterization of different components of wastewater, sludge and wastewater (Alves Filho et al. 2015). In characterization of fulvic and humic acids in wastewater and sludge, the ^1^H NMR spectra are dominated by broad unresolved humps characteristic of complex mixtures, with some sharp signals indicative of specific molecules or structures (Alves Filho et al. 2015; Ma et al. 2001; Polak et al. 2005). Due to the complexity of such matrices, the identification of each molecule is not possible, and, as reported in previous works on the characterization of fulvic and humic acids in soil and sludge (Gigliotti et al. 2003; Alves Filho et al. 2015; Polak et al. 2005), the ^1^H NMR spectra are divided into different major resonance areas in order to characterize the main functional groups and structures such as aliphatic and aromatic compounds.

In this work, we have applied ^1^H NMR spectroscopy to characterize the main functional groups, before and after treatment, and to follow the degradation of the main organic compounds. We have selected three different regions: 0–3.0 ppm, corresponding to alkylic chains and aliphatic compounds; 3.0–6.0 ppm, corresponding to compounds bearing groups such as –OH, –NH_2_, alkyl halides and alkenes; and 6.0–9.0 ppm corresponding to aromatic protons. For each major resonance area, the sum of the ^1^H integral, for pure leachate and after treatment, has been calculated to monitor the modification of the degradation of organics. In summary, ^1^H NMR analysis resulted to be useful for monitoring the efficiency of the treatment process by specifically tracing the abatement of aromatic compounds which are mainly responsible for toxicity and are the most persistent toward oxidation reactions in liquid waste.

Usually, catalyst performance over complex waste processes is monitored through the evaluation of TOC/COD (Total Organic Carbon/Chemical Oxygen Demand) abatement (Ribeiro & Nunes 2021), but the calculation of these parameters cannot disclose the overall treatment potentiality. In addition to the degree of mineralization/oxidation, to better monitor the detoxification of the treated liquid waste, it is necessary to improve our understanding on the removal of organics. In this study, the abatement of representative classes of compounds (aromatics and AOX) has been measured and correlated to the toxicity to find a simple preliminary approach for the qualitative assessment of the reduction of the ecotoxicological risk.

## Materials and methods

Landfill leachate samples were collected in polyethylene bottles from a landfill site located in Friuli Venezia Giulia, Italy, and were preserved at 4 °C. The preparation of the copper supported over zirconia (5 wt% Cu/ZrO_2_) and the catalytic activity experiments were carried out as previously described by Hussain et al. (Hussain et al. 2020). Briefly, 5% Cu/ZrO_2_ was prepared by incipient wetness impregnation of ZrO_2_ (MEL Chemicals) with an aqueous solution of copper (II) nitrate hemi pentahydrate (Sigma-Aldrich). After impregnation the material was dried overnight at 100 °C and calcined for 3 h at 500 °C.

### Catalytic activity measurements and qualitative characterization of landfill leachate

#### Catalytic activity

The leachate samples (100 mL) loaded with the 200 mg/L of ZrCu catalyst were heated at 70 ˚C, pH 5 in the presence of 200 mg/L of catalyst, under reflux and continuous stirring conditions (500 rpm) using an Omni multi stage reaction station. Finally, 30 mL/L of hydrogen peroxide solution (3% w/w in H_2_O) was added into the reaction system, and the Fenton-like oxidation reaction was carried out for 150 min.

The treated samples were centrifuged at 5000 rpm using Eppendorf Centrifuge 5804 R for 10 min, and the supernatant solutions were collected in vials for TDOC and COD analysis. Total dissolved organic carbon (TDOC) was used instead of total organic carbon (TOC), because the suspended organic matter in leachate may affect the oxidation process.

The TDOC of the treated samples was analyzed using a TOC-VCPN, Shimadzu analyzer (V-Series) with an autosampler. First, the standard solutions with known concentrations of 2.5, 5.0, 10.0, 20.0 and 50.0 mg C/l were prepared using potassium hydrogen phthalate stock solution. A calibration curve was obtained by analyzing the standard samples, and after each analysis, the injection syringe was auto sparged with 2 M HCl. The samples were analyzed without dilution and using the same calibration curve to determine the TDOC present in each sample.

To determine the chemical oxygen demand (COD) of samples, 2 mL of distilled-deionized water (blank reference) and leachate samples were separately added into COD testing kits. These testing kits were thoroughly shaken and subjected to heating at 150 °C for 2 h using the HACH COD reactor. Later, these vials were cooled down to room temperature, and the COD values were measured using a spectrophotometer (PC MULTIDirect).

All the experiments were conducted in triplicate to verify the reproducibility of our activity measurements, and the errors resulted to be within 3% for TDOC and 10% for COD measurements.

#### UV–Vis spectrophotometric analysis

UV–Vis analysis of samples is carried out using Shimadzu 2600i spectrophotometer, and absorbance was measured from 200 to 700 nm, in a quartz cell with a path of 1 cm. Each wavelength on the UV–vis spectra corresponds to the concentration of a particular organic species especially in the range from 220 to 270 nm where most of the aromatics exhibit their maximum absorptions (GilPavas et al. 2019).

#### AOX analysis

To measure the concentration of adsorbable organic halogens (AOX), the leachate samples were first diluted 1/100 times with distilled-deionized water and mixed with 1 g/L of activated carbon. To purge the chlorides coming from inorganic sources, 1 mL of 0.1 M solution of Na_2_S_2_O_3_ was added into the solution, and the mixture was subjected to shaking at 180 rpm/m using KS 501 D Shaker (IKA-Werke GmbH & Co. KG) for 1 h. The thoroughly mixed solution was then filtered through 0.2-µm cellulose acetate membrane filter, followed by washing the filter cake with 0.5 M NaNO_3_ solution to eliminate any residual inorganic sourced chlorides. The membrane filter carrying activated carbon was inserted into the Euroglass AOX sampler, and the concentration of AOX was measured. Several experiments were also carried out to verify the reproducibility of our measurements, and the errors resulted to be within 5%.

#### ^1^H NMR spectroscopic analysis

A volume of 200 µL of raw and treated landfill leachate samples was separately dissolved in 800 µL of D_2_O followed by thorough mixing. The samples were subjected to ^1^H NMR analysis using Bruker AvanceIII HD 400 MHz NMR spectrometer. NMR spectra were acquired employing a gradient-based water suppression pulse sequence with acquisition time of 3 s and 128 scans. After Fourier transformation, the spectra were phase- and baseline-corrected manually.

The qualitative efficacy in the organic abatements over the Fenton-like process is determined by analyzing the resonance signals in the spectra of landfill leachate before and after treatment.

#### Toxicity test with green algae

The algal growth inhibition test with *Raphidocelis subcapitata* was performed in a 24-well plate following the OECD 201 guideline and ISO 8692:2012 as previously reported (Croce et al. 2017). An aliquot of cryofrozen algae (1 million/vial) was taken from the biobank and cultured in BG-11 medium at 25 ± 1 °C in an incubator in the presence of light. Before use in the ecotoxicological test, the algae were kept in line for 2 weeks with a bi-weekly renewal of the culture medium to allow the exponential growth of the algal population. After 14 days, the response of the algal population to potassium dichromate in the range 0.1–3.2 mg/L was evaluated as an indicator of the suitability of the culture for use in the toxicity test. Once the desired sensitivity was reached, the algal growth inhibition assay was performed. The algae (10,000 cells/mL) were inoculated into the wells of the multiwell plate containing dilutions of the leachate as it is or treated in previously oxygenated reconstituted standard water (ISO Algal Freshwater). The reconstituted standard water was used as negative control, while the potassium dichromate (range 0.1–3.2 mg/L) was used as a positive control to check algal sensitivity during the test. The multiwell plate was then incubated at 23 °C for 72 h in the presence of light and under dynamic shaking (orbital shaker, 90 rpm). At the end of the exposure period, the algal growth in each well was determined as the change in the number of algae per mL with respect to time 0 (inoculum) by means of a TC20™ Automated Cell Counter (Bio-Rad Laboratories, Inc), selecting a dimensional gate between 4 and 10 microns. Results were expressed in terms of biomass and growth rate.

## Results and discussion

Textural, structural and redox characteristics of the catalyst have already been reported (Hussain et al. 2020). The surface area is 55 m^2^/g, the material is composed of tetragonal and monoclinic zirconia and copper is homogeneously dispersed on the surface of the support. The landfill leachate used in the study is in its intermediate phase, and it is characterized by an initial COD of 8700 mg/L, TDOC of 970 mg/L, pH 8.5 and a low concentration of heavy metal ions (Hussain et al. 2022).

The heterogeneous Fenton process was investigated on ZrCu catalyst performing reactions at 70 °C, for 150 min with 200 mg/L of catalyst, 30 mL/L of H_2_O_2_ at pH 5. The abatement of the organics has been evaluated by means of TDOC and COD parameters. TDOC indicates the degree of mineralization (complete conversion to CO_2_ and H_2_O), while COD is related to the degree of oxidation (degradation of organic compounds into more oxidized molecules). The treatment results in a similar abatement in TDOC and COD of around 65%, (COD/TOC ≈ 1) indicating that a strong mineralization occurs during the treatment (Hussain et al. 2022). The degradation of the organics in the leachate is very fast, and the higher abatement is achieved in 30 min. The influence of reaction variables has been investigated in a previous paper (Hussain et al. 2022). This is an important target because the main aim of the reaction is to achieve the highest degree of mineralization, but it is also essential to evaluate the overall toxicity of the landfill leachate after treatment. The oxidation process, indeed, should not follow a reaction pathway that leads to the formation of more toxic metabolites than the parent organic substances. Therefore, in this work, the qualitative efficacy of the process has been investigated through several techniques (UV, ^1^H NMR and AOX analysis) and compared with bioassay risk assessment tests to find a preliminary tool for the evaluation of the effective reduction of the ecotoxicological risk after the treatment.

### Analysis of the qualitative efficacy of the Fenton-like process

**Fig. 1:**
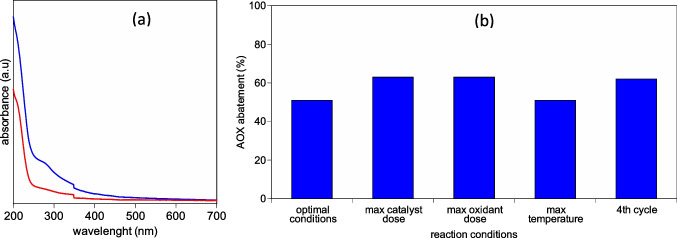
**a** UV–Vis spectrum of leachate before (blue line) and after treatment (red line); **b** AOX abatements with variable process conditions. (Optimal conditions: 70 °C, pH 5, catalyst dose 200 mg/L, H_2_O_2_ 30 mL/L and reaction time 150 min; max catalyst dose 300 mg/L; max oxidant dose 50 mL/L; max temperature 80 °C)

Landfill leachate is a very complex mixture of molecules with thousands of different compounds, and the targeted quantification of organic substances is extremely difficult. For this reason, the bulk composition is largely unexplored due to analytical limitations, while general characterization and functional group identification are usually performed. The qualitative abatement of organics, and specifically aromatics and AOX, can give some further insight into the efficacy of the Fenton process in the reduction of the toxicity of the liquid waste. Landfill leachate samples, before and after treatment, have been analyzed by using UV–Visible spectroscopy and absorbable organic halogens (AOX) analysis (Fig. 1).

The heterogeneous Fenton-like oxidation has markedly reduced the concentrations of organic compounds which can be observed in terms of substantial reduction in absorptions (Fig. 1A). Each wavelength on the spectra corresponds to the concentration of a particular organic species especially in the range from 220 to 270 nm where most of the aromatics exhibit their maximum absorptions (GilPavas et al. 2019). Specifically, absorbance at 254 nm (UV 254) is usually recognized as an indicative parameter to evaluate the level of aromatic contents (Chen et al. 2018). A significant decrease of absorbance at 254 nm, from 0.456 to 0.169, is observed for treated leachate, suggesting a lowering of the aromatics. In addition, the A_210_/A_254_ ratio is well correlated to the presence of aliphatic/aromatic compounds, where low values of A_210_/A_254_ indicate a predominantly aromatic nature (Trubetskaya et al. 2020), and consequently it can be applied for the characterization of the leachate before and after treatment. The A_210_/A_254_ ratio is 3.5 for the leachate before the treatment and increases considerably after the reaction (5.8) indicating an important reduction of the aromatic compounds due to the oxidation process.

Absorbable organic halogens (AOX) are one of the most persistent organic pollutants. Their concentration must be controlled in liquid waste due to adverse environmental impacts (Lohmann et al. 2007; Qin et al. 2018), and many countries have set a limit for their concentration in wastewater streams; consequently, their analysis in the leachate is essential (Balabanič et al. 2017; Xie et al. 2018). The Fenton-like process over ZrCu, carried out under conventional conditions (reaction condition: 70 °C, pH 5, catalyst dose 200 mg/L, H_2_O_2_ dose 30 mL/L, and reaction time 150 min), achieved a reduction of 51% of AOX (Fig. 1b). The increase in catalyst (300 mg/L) and oxidant dose (50 mL/L) induces a higher AOX removal efficacy (63%), while the increase in the reaction temperature (80 °C) did not significantly affect the AOX abatement. Remarkably, AOX reduction remains stable after 4^th^ cycles of reuse of the catalyst, suggesting that ZrCu is a very stable and promising catalyst for the detoxification of the liquid waste.

**Fig. 2:**
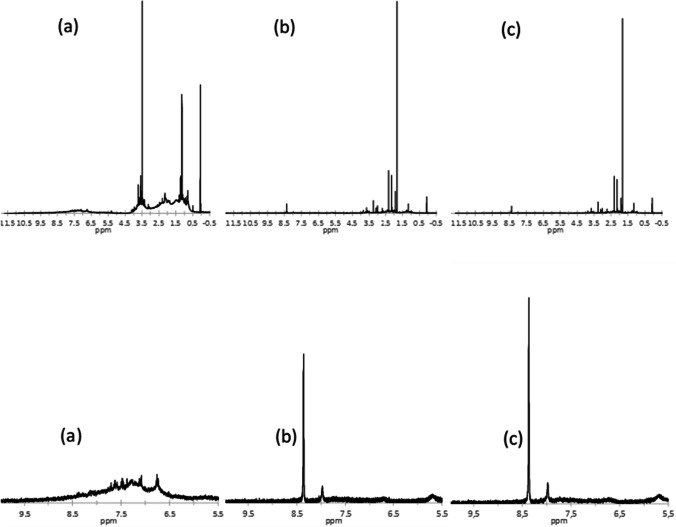
^1^H NMR spectrum of landfill leachate before treatment (**a**) after 30 min (**b**) and 150 min (**c**). At the bottom, the detail of the region 5.5**–**10 ppm

Here, for the first time, ^1^H NMR spectroscopic analysis has been applied to monitor the qualitative abatement of contaminants from landfill leachate. ^1^H NMR not only provides insight into the nature of the organic compounds present in the leachate but is equally useful to understand the qualitative efficacy of the Fenton-like oxidation. The ^1^H NMR spectrum of the leachate (Fig. 2) can be divided into three major resonance areas: the 0–3.0 ppm area, mainly due to alkylic chains and aliphatic compounds, the 3.0–6.0 ppm range, typical for compounds bearing groups such as –OH, –NH_2_, alkyl halides and alkenes, and the 6.0–9.0 ppm region characteristic for resonances of aromatics compounds.

All three regions are characterized by broad unresolved resonance with overlapping well-defined peaks. The broadness of these peaks indicates a significant overlap of signals from multiple protons with the same resonant frequencies, consistent with a complex mixture of compounds.

**Fig. 3:**
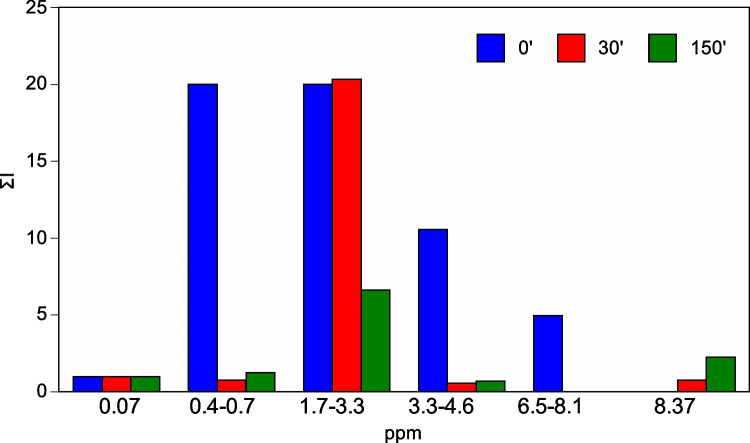
Comparison of the ^1^H integral of the main regions appearing in.^1^H NMR spectrum in pure leachate and after treatment (30 and 150 min of reaction)

The ^1^H NMR spectra of the treated leachate (at 30 and 150 min) appear to be completely modified; in particular broad, unresolved resonances are drastically reduced and are mainly found relatively well-resolved peaks or clusters of peaks. These results reveal that the Fenton-like process affects all these three resonance regions leading to a significant simplification of the spectrum. As can be seen, the initial complex mixture is now reduced to a mixture of a small number of compounds, some of which can be tentatively recognized, based on the chemical shift, as acetic acid (2.1 ppm), acetone (2.3 ppm) and formic acid (8.3 ppm). In particular, the 4.5–9 ppm region shows only the formic acid signal (8.3 ppm) being the original aromatic signals completely disappeared, while the 0–4.5 ppm region is dramatically simplified, suggesting that the ZrCu catalyst is extremely promising for the degradation of organics. Placing the 0.07 ppm signal at 1, the sum of the ^1^H integral (ΣI) in the main regions appearing in spectra before and after treatment (at 30 and 150 min) were compared (Fig. 3). After treatment, a significant decrease is observed in the regions 0.4–0.7, 3.3–4.6 and 6.5–8.1 compared to pure leachate, while in the range 1.7–3.3, the integral is almost the same after 30 min and decreases after 150 min of reaction.

As detailed in Fig. 2, the signals, found before and after reaction, in the same regions, are due to different compounds. These differences in the NMR analysis indicate a transformation of aromatic and non-aromatic molecules toward oxidized compounds. The two spectra at 30 and 150 min appear very similar confirming that the reaction is fast and ZrCu degrades the organic matter of the leachate in the first 30 min, and in additional reaction times progressive oxidation of the secondary organic molecules occurs, pushing toward simpler compounds such as formic acid, increasing the biodegradability of the liquid waste. To summarize, these outcomes indicate that the oxidation process significantly affects the overall organic removal efficiency and specifically is very active in the degradation of aromatics compounds.

### Bioassays for ecotoxicological risk assessment

A study on the effects of leachate on unicellular green algae was carried out under controlled laboratory conditions according to validated protocols. The study evaluated the toxicity of leachate pre- and post-mineralization treatment as effects inhibiting the proliferation of a population of unicellular freshwater green algae, used for years as a model organism of algae and the trophic level of producers (plants, algae, photosynthetic bacteria) in ecotoxicity studies for the aquatic environment (Ghosh et al. 2017). Bioassays provide a measure of the overall toxicity of the investigated matrix responding to all their chemical constituents and accounting for the bioavailability and the interactions of all the agents (Baderna et al. 2019; Farré and Barceló 2003; Ghosh et al. 2017).

In the present study, the antiproliferative effects of 2 leachate samples (leachate as it is and after Fenton treatment) on the algal population of *R. subcapitata* were investigated. The samples were studied in concentrations from 0.1 to 20% volume/volume, by directly diluting the samples with standard reconstituted water. It was not necessary to modify the pH of the samples as they had values compatible with the survival of the algae (between 8.1 and 8.4). The results of the study are reported in Table 1 relating to biomass and growth rate.

**Table 1:** Algal growth inhibition test. The results are normalized on the control and the analysis based on the change in algal density (biomass) and growth rate

Sample	% leachate v/v	Biomass		Growth rate	
		% CTR	% STD	% CTR	% STD
Before treatment	0	100.00	4.80	100	0.99
	0.1	83.52	1.95	96.49	0.47
	0.5	4.43	1.11	38.56	12.39
After treatment^a^	0	100.00	4.80	100	0.99
	0.1	89.64	5.10	97.85	1.14
	0.5	79.67	10.57	95.44	2.75
	1	28.99	2.73	75.69	2.56
	2.5	20.59	1.22	69.03	1.69
	5	3.74	0.12	35.61	1.71

^a^Reaction conditions: 70 °C, pH 5, catalyst dose 200 mg/L, H_2_O_2_ dose 30 mL/L and reaction time 150 min

The study showed that the leachate is a very toxic sample and that the maximum effect is reached already at the dilution of 0.5%, while at 0.1, the lowest point tested, an inhibition of about 15% is obtained compared to the control. The inhibitory effect of 50% is obtained at an estimated concentration of 0.17% by evaluating the biomass and at 0.49% by evaluating the growth rate. The Fenton treatment substantially changes the toxicity of the leachate. The inhibitory effect of 50% is obtained at an estimated concentration of 0.80% considering the biomass and 3.25% considering the growth rate. These results are of fundamental importance; indeed, the focus of the catalytic treatment is not only the mineralization of the leachate but also its detoxification. These results indicate detoxification of the liquid waste after Fenton treatment, suggesting that the process can completely mineralize a part of the organic compounds and partially degrade other pollutants into less toxic molecules, in perfect agreement with the qualitative characterization of the treated liquid waste.

UV–vis spectroscopy, AOX and ^1^H NMR analysis indicate an important reduction of the organic contaminants in the leachate after the Fenton treatment over ZrCu, with a significant abatement of the aromatic and halogen compounds (51%) that represents the most toxic fraction. The qualitative evaluation obtained by coupling these techniques can suggest, as a first approximation, the efficiency of the treatment in the detoxification of the leachate.

While the ecotoxicological test is resource-intensive and extremely time-consuming, coupling AOX, UV–visible and ^1^H NMR analysis are fast and simple, reducing the time and complexity for a first evaluation of the effectiveness of an advanced oxidation treatment.

Therefore, according to the above results, the more accurate and time-consuming ecotoxicological test could only be reserved to the catalytic formulation or reaction conditions that shows the most promising results in Fenton-like processes.

## Conclusion

This study, for the first time, has successfully suggested a new way of monitoring the qualitative performance of Fenton reaction using H^1^ NMR. Furthermore, it highlights the possibility of using the qualitative abatement of specific classes of compounds such as aromatic and halogenated organics by means of ^1^H NMR, UV and AOX analysis as a fast and simple preliminary tool for a first approximation of the reduction of the ecotoxicological risk reserving the more complex toxicity test for the most promising catalytic processes. The qualitative abatement of aromatics by spectroscopy analysis and the reduction of AOX compounds (51%) indicates a significant reduction of the toxicity of the leachate, validated by ecotoxicological test with algae, confirming ZrCu as a promising catalyst for the Fenton-like treatment of heavily polluted liquid waste.

## Data Availability

Not applicable.
